# Interventions to improve medicines optimisation in frail older patients in secondary and acute care settings: a systematic review of randomised controlled trials and non-randomised studies

**DOI:** 10.1007/s11096-021-01354-8

**Published:** 2021-11-20

**Authors:** Dima Saeed, Gillian Carter, Carole Parsons

**Affiliations:** 1 School of Pharmacy, Queen’s University Belfast, Belfast, UK; 2 School of Nursing and Midwifery, Queen’s University Belfast, Belfast, UK

**Keywords:** Frailty, Frail elderly, Medicines optimisation, Medication review, Secondary care, Systematic review

## Abstract

*Background*: Frailty is a geriatric syndrome in which physiological systems have decreased reserve and resistance against stressors. Frailty is associated with polypharmacy, inappropriate prescribing and unfavourable clinical outcomes. *Aim*: To identify and evaluate randomised controlled trials (RCTs) and non-randomised studies of interventions designed to optimise the medications of frail older patients, aged 65 years and over, in secondary or acute care settings. *Method*: Literature searches were conducted across seven electronic databases and three trial registries from the date of inception to October 2021. All types of interventional studies were included. Study selection, data extraction, risk of bias and quality assessment were conducted by two independent reviewers. *Results*: Three RCTs were eligible for inclusion; two employed deprescribing as the intervention, and one used comprehensive geriatric assessment. All reported significant improvements in prescribing appropriateness. One study investigated the effect of the intervention on clinical outcomes including hospital presentations, falls, fracture, quality of life and mortality, and reported no significant differences in these outcomes, but did report a significant reduction in monthly medication cost. Two of the included studies were assessed as having ‘some concerns’ of bias, and one was judged to be at ‘high risk’ of bias. *Conclusion*: This systematic review demonstrates that medicines optimisation interventions may improve medication appropriateness in frail older inpatients. However, it highlights the paucity of high-quality evidence that examines the impact of medicines optimisation on quality of prescribing and clinical outcomes for frail older inpatients. High-quality studies are needed to address this gap.

**Supplementary Information:**

The online version contains supplementary material available at 10.1007/s11096-021-01354-8.

## Impact on practice

- Medicines optimisation interventions are safe, feasible and effective in improving the appropriateness of prescribing among frail hospitalised older patients.
- Hospitalised older patients should be screened for frailty using a validated instrument to inform the implementation of effective interventions in the context of the individual’s morbidities, goals of care and life expectancy.
- Medication review should be conducted regularly in frail older patients to evaluate whether medicines are providing ongoing net benefit or net harm.

## Background

Frailty is a syndrome characterised by a cumulative decline across multiple physiological systems as well as decreased reserve and resistance against internal and external stressors; any small stress or minor illness may trigger a dramatic change in health status [1–3]. Frailty phenotype (FP) and Frailty index (FI) are the two major frailty models [3–5]. FP identifies frailty by the presence of three or more of five phenotypic criteria weakness, slowness, low level of physical activity, self-reported exhaustion, and unintentional weight loss [6]. FI defines frailty as cumulative age-related health deficits including physical, psychological, and social deficits [7]. About one-quarter of the population aged ≥ 50 years across the world is frail [8]. Frailty is associated with unfavourable clinical outcomes including falls, hospitalisation, and mortality [1, 2, 9, 10].

Polypharmacy (the concurrent use of multiple medications) and potentially inappropriate prescribing (PIP) are highly prevalent among frail older patients who are at greater risk of experiencing more severe and frequent drug-related negative outcomes than robust older adults [10–14]. PIP incorporates ‘overprescribing’ (the use of drugs without a valid indication), ‘misprescribing’ (the utilisation of incorrect drug, dose or route of administration) [15, 16] and ‘underprescribing’ (the omission of a potentially beneficial medication clinically indicated for the prevention of disease) [17–20], and refers to the prescribing of potentially inappropriate medications (PIMs), defined as medications with questionable efficacy that may cause significant risk of adverse drug reactions, excess morbidity and mortality [15, 21, 22].

Acute admissions present valuable opportunities to review and optimise medicines for frail older people and to deprescribe PIMs in the context of an individual’s morbidities, goals of care and life expectancy [11]. Medicines optimisation is “a person-centred approach to safe and effective medicines use, to ensure people obtain the best possible outcomes from their medicines” [23]. A regular medication review for frail patients allows evaluation of whether medications are providing ongoing net benefit or net harm [11, 24, 25]. Medication review is defined as a systematic and structural evaluation of a patient’s pharmacotherapy to optimise medication use by identifying the medication-related problem and then changing the prescription [26, 27]. Targeted deprescribing among vulnerable populations is an important element of optimising medication. Deprescribing is defined as a process of addressing and stopping inappropriate medication. It encompasses medication withdrawal and reduction of medication dose and frequency [28–32]. A recent systematic review demonstrated that deprescribing among older people living with frailty in all types of healthcare settings significantly reduced the number of prescribed medications and PIMs [33]. However, little work has been conducted in hospitalised frail older patients to examine the impact of medicines optimisation interventions in secondary and acute care settings.

## Aim

This systematic review aimed to identify and evaluate studies of interventions designed to optimise the medications of frail patients aged ≥ 65 years in secondary or acute care settings and identify medication- and patient-related outcomes related to medicines optimisation reported in such studies.

## Method

This systematic review was conducted in accordance with PRISMA (Preferred Reporting Items for Systematic Reviews and Meta-Analyses) 2020 guidelines [34] (Supplementary Material 1). The protocol was registered in the International Prospective Register of Systematic Reviews (PROSPERO) registry (registration number CRD42019156623) [35]. This review presents further analysis and discussion of preliminary findings presented previously [36].

## Search strategy

Comprehensive literature searches were conducted to identify eligible studies and ongoing or completed clinical trials, published in English, from the date of inception to 13th October 2021. Seven electronic databases and three trial registries were searched: Medline, Scopus, Embase, Web of Science, International Pharmaceutical Abstracts, Cumulative Index to Nursing and Allied Health Literature Plus (CINAHL Plus), Cochrane Library, Cochrane Central Register of Controlled Trials, ClinicalTrials.gov, International Clinical Trials Registry Platform and Research Registry. According to the database being searched, search terms used were keywords, medical subject headings (MeSH) and EMTREE headings (Elsevier Life Science Thesaurus). Search terms were developed and refined with the assistance of a Queen’s University Belfast subject librarian (search strategy is detailed in Supplementary Material 2). The reference lists of eligible studies were also hand-searched for any additional suitable articles that may have been missed during the database searches.

## Study selection and eligibility criteria

All types of intervention studies aiming to optimise medications use in frail older inpatients (aged ≥ 65 years) were eligible for inclusion in this review. The inclusion criteria for this review were guided by the population, intervention, comparator, outcome (PICO) framework [37], as outlined in Table 1.

**Table 1:** Inclusion criteria for eligible studies

Population	• Frail older patients aged 65 years and over in secondary or acute care settings • Frailty diagnosis using any existing frailty assessment tool
Interventions	• Intervention relating to any aspect of ‘Medicines Optimisation’, ‘Medicines Management’, ‘Pharmaceutical care’, ‘Medication Review’ or ‘Deprescribing’ •Interventions delivered by any healthcare professional including geriatricians, pharmacists, nurses, or by a multidisciplinary team
Comparators	• Frail older inpatients (aged ≥ 65 years) receiving: a. Usual care (care as usually received by patients in everyday practice) or b. No service (no intervention provided)
Outcomes	• Any change in medication (dose, frequency, dosage form, number of medications stopped or started) • Appropriateness of prescribing • Adverse drug reactions • Death • Quality of life • Falls or recurrent falls • Fractures • Disability • Cost of medication and/or cost of health care utilisation (i.e.hospital readmission and duration of hospitalisation)
Study design	• All types of randomised controlled trials (RCTs) • Non-randomised studies (NRSs)

Patients diagnosed with pre-frailty were not eligible for inclusion. Pre-frailty is an early and reversible risk-state before frailty [38] and is identified using the FP model by the presence of one or two of the five phenotypic criteria [6]. Additionally, multicomponent intervention studies where it was not possible to determine which component of the intervention was responsible for the reported outcomes were also excluded.

## Study selection

After removing duplicates, initial assessment of the article titles and abstracts was conducted by the researcher (DS); studies that did not meet the inclusion criteria were excluded. Two reviewers (DS and CP) independently reviewed the full text of all potentially eligible articles to determine if they met the inclusion criteria. Both reviewers discussed their results and a third reviewer (GC) was consulted if consensus could not be reached regarding including or excluding a study. A PRISMA 2020 flow diagram was generated to display the screening process and reasons for inclusion and exclusion of studies [34].

## Data extraction

Data extraction was performed independently by two review authors (DS and CP) using the Cochrane data collection form as a template [39]. In the case of discrepancies, a third researcher (GC) was consulted. Study authors were contacted if data were missing.

## Quality assessment

Two review authors (DS and CP) independently assessed the risk-of-bias of each study using the Cochrane Collaboration’s Risk of Bias (ROB 2.0) tool for RCTs [40]. Disagreements were resolved by consensus and a third reviewer (GC) was consulted where necessary.

## Results

### Study selection

**Fig. 1:**
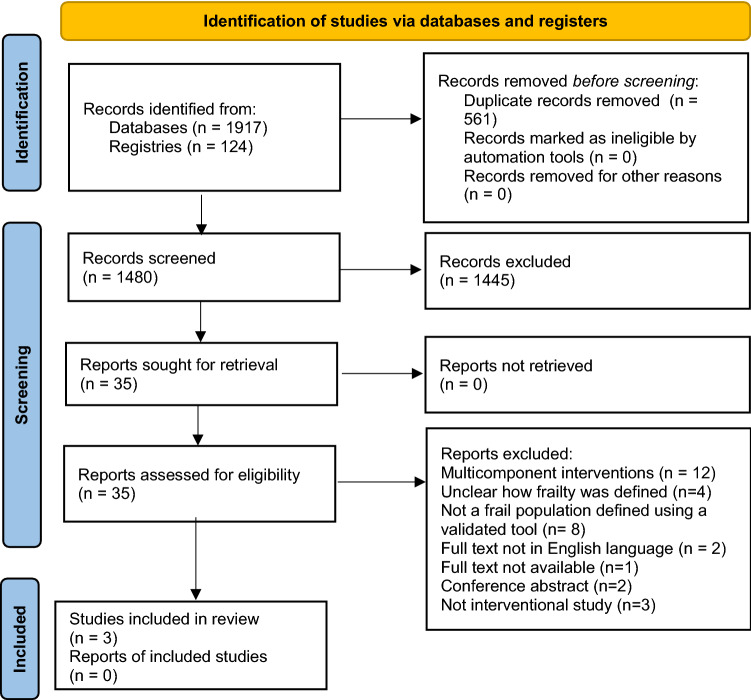
Preferred Reporting Items for Systematic Reviews and Meta-Analyses (PRISMA 2020) flow diagram

A total of 2041 articles were retrieved. After removal of duplicate publications, 1480 articles were screened for eligibility based on their titles and abstracts; full texts of 35 articles were assessed for eligibility. Of these, three RCTs were deemed eligible for inclusion [41–43]. Further studies were not identified from a manual search of the references of included studies. The study selection process and reasons for exclusion are summarised in Fig. 1, and the full list of excluded studies and reasons for exclusion are provided in Supplementary Material 3.

## Study characteristics

The characteristics of the included studies are summarised in Table 2. Due to the heterogeneity of the included studies, meta-analysis was not possible, and a narrative synthesis was conducted.

**Table 2:** Characteristics of included studies on the impact of medicines optimisation on frail older people in acute and secondary care settings (published between 2004 and 2020)

Study ID (country)	Sample size (Age of participants)	Frailty assessment	Intervention provider (Follow- up period)	Description of intervention	Measured outcomes and Findings
Schmader et al., 2004 (USA) [41]	834 patients (≥ 75 years)	1. Frail patients who met two or more of the following criteria: a. inability to perform one or more basic activities of daily living b. stroke within the previous three months c. a history of falls d. difficulty walking, e. malnutrition f. dementia g. depression h. one or more unplanned admission(s) in the previous three months i. prolonged bed rest j. incontinence	Core team including a geriatrician, social worker, nurse and pharmacists (12 months)	Intervention group: inpatient geriatric evaluation and management according to published guidelines and VA standards Control group: Usual care	1. Suboptimal prescribing; number of unnecessary drugs, inappropriate prescribing, and underuse a. The reduction in the no. of unnecessary drugs between baseline and discharge was significantly greater in intervention group compared to control group; difference in change (95% CI): −0.5 (−0.7 to −0.4) (P-value ˂0.0001) b. The improvement in the MAI score between baseline and discharge was significantly greater in intervention group compared to control group; difference in change (95%CI): −5.4 (−6.5 to −4.3) (P-value ˂0.0001) c. The reduction in no. of PIMs (as demonstrated by Beers criteria) between baseline and discharge was significantly greater in intervention group compared to control group; difference in change (95%CI): −0.1 (0.2 to −0.01) (P-value = 0.03) d. The reduction in no. of conditions with omitted drugs between baseline and discharge was significantly greater in intervention group compared to control group; difference in change (95%CI): −0.3(−0.5 to −0.1) (P-value ˂0.0001) 2. Adverse drugs reactions a. All type of adverse drug reactions were detected more frequently in intervention group; RR (95% CI): 1.85 (1.40 to 2.45) (P-value = 0.0001) b. Serious adverse drug reactions did not significantly differ between intervention and control group; RR (95% CI): 1.03 (0.55 –1.95) (P-value = 0,93)
Dalleur et al., 2014 (Belgium) [42]	158 patients (≥ 75 years)	1. ISAR score of ≥ 2/6 (one point for each of the following: a. needing help with activities of daily life b. an increase in this need related to the current illness c. memory problems d. significantly altered vision e. hospitalisation in the previous 6 months f. daily use of three or more medications at home	IGCT consisting of nurses, geriatricians, a dietician, an occupational therapist, a physiotherapist, a speech therapist, and a psychologist (12 months)	Intervention group: STOPP recommendations made by the IGCT to ward physicians to discontinue PIMs, in addition to standard geriatric advice Control group: Standard care, comprising routine medication review by the IGCT geriatrician, using an implicit approach (i.e. no explicit tool used)	1. Proportion of PIMs discontinued (or corrected in case of dose-related or duration-related PIMs) between hospital admission and discharge (according to discharge letter) a. PIM discontinuation rate in intervention group (39.7%) was significantly greater than in control group (19.3%); OR (95% CI): 2.75 (1.22 to 6.24) (P-value = 0.013) b. Proportion of patients with at least one improvement to their drug treatment significantly higher for intervention group than for control group (25.7% vs 13.9% respectively) (P-value 0.034)
Curtin et al., 2020 (Ireland) [43]	130 patients (≥ 75 years)	1. Severely frail a. CFS of 7 or higher b. Less than one-year life expectancy	Physician (3 months)	Intervention: Usual pharmaceutical care supplemented by individualized STOPPFrail-guided deprescribing plan Control group: usual pharmaceutical care	1. Mean change in no. of long-term prescribed medicines from baseline to 3 months after randomisation a. Significantly greater decrease in no. of long-term prescribed medications at 3 months in intervention group compared to control group; mean difference = 2.25 ± 0.54; 95% CI = 1.18 to 3.32 (P-value < 0.001) 2. Hospital presentations a. No significant difference in the no. of ED visits between intervention and control groups; RR (95% CI) 0.60 (0.15 to 2.41) (P-value = 0.72) b. No significant difference in no. of unplanned hospital admissions; RR (95% CI) 1.80 (0.64 to 5.08) (P-value = 0.27) 3.No significant difference in no. of falls between intervention and control groups; RR (95% CI) 0.90 (0.48 to1.69) (P-value = 0.75) 4. No significant difference in no. of non-vertebral fractures between intervention and control groups; RR (95% CI): 0.23 (0.03 to 1.95) (P-value = 0.18) 5. Changes in participants’ quality of life a. No significant difference in quality of life of participants between interventions and control groups as demonstrated by ICECAP-O; mean difference (95% CI) = −0.09 (0.04 to 0.21) (P-value = 0.17) b. No significant difference in quality of life of participants between intervention and control groups as demonstrated by QUALIDEM; mean difference (95%CI) = 0.37 (−2.03 to 1.19) (P-value = 0.60) 6. No significant difference in mortality between intervention and control groups; RR (95% CI): 0.67 (0.35 to 1.27) (P-value = 0.22) 7. Deprescribing contributed to medication cost savings; mean change in monthly medication cost at 3-month follow-up as significantly higher in intervention group compared to control group; mean difference (95% CI) = $61.74 ± $26.60 (8.95 to 114.53) (P-value = 0.02)

*CFS*: Clinical Frailty Scale, *CI*: Confidence interval, *ED*: Emergency department, *ISAR*: Identification of Seniors at Risk, *IGCP*: Inpatient Geriatric Consultation Team, *MAI*: Medication Appropriateness Index, *OR*: odds ratio, *PIM*: potentially inappropriate medication, *QUALIDEM*: Quality of life in dementia scale, *RCT*: Randomised controlled trial, *RR*: Relative Risk, *STOPP*: Screening Tool of Older Person’s Prescriptions, *VA*: Veterans Affairs, *No*.: Number

## Participants

A total of 1133 hospitalised patients were recruited across the three included studies (mean sample size 378 participants, range 130–845). Curtin et al., explicitly targeted frail patients at the end of life and used the surprise question, whereby the clinician indicated that he or she "would not be surprised if the patient died in the next year”[44], to indicate one-year life expectancy [43].

## Characteristics of interventions

The interventions in the included studies were delivered by a multidisciplinary team (n = 2) [41, 42] or a physician (n = 1) [43]. The deprescribing interventions used a specific explicit tool in two studies; one [42] employed the Screening Tool of Older Person's Prescriptions (STOPP) criteria [20] and the other [43] employed the STOPPFrail criteria [45]. The third study employed geriatric evaluation and management according to published guidelines and Veterans Affairs (VA) hospital standards, whereby a comprehensive medication review was undertaken to assess all aspects of suboptimal prescribing (overuse, misuse, and underuse) and a combination of implicit and explicit PIM screening tools was utilised [41] including the Medication Appropriateness Index (MAI) [46], Beers Criteria [47] and the Assessment of Underutilization of Medication instrument [48].

## Effect of interventions: outcomes

All included studies reported significant improvements in prescribing appropriateness [41–43]. Different outcomes were used as measures of prescribing appropriateness across the included studies, and included number of prescribed medications (n = 1) [43] and PIM discontinuation rate (n = 1) [42]. The third study reported several outcomes to cover all aspects of suboptimal prescribing, including the number of unnecessary medications, MAI score, number of PIMs according to the Beers criteria and number of conditions with omitted drugs [41] (Table 1). Two studies reported changes in prescribing of specific drug classes; one reported changes in prescription of antipsychotic medications and found that antipsychotic drugs were discontinued more often in intervention patients relative to control patients, however, the difference did not reach statistical significance [43]. The second study reported PIM discontinuation rate for the most commonly deprescribed drug classes benzodiazepines, antiplatelets, opioids, ß-blockers, tricyclic antidepressants and neuroleptics and found that the discontinuation rate was higher in the intervention group than in the control group. However, the *P* value was only reported for benzodiazepines (*P* value = 0.0063) [42].

Schmader et al. investigated the effect of medication review and optimisation on adverse drug reactions (ADRs) and reported that the number of all ADRs (minor plus serious) was significantly higher in the intervention group. However, there was no significant difference between intervention and control groups regarding the risk of serious ADRs.

Curtin et al. outlined the impact of deprescribing on health-related outcomes for frail older people after discharge, including quality of life (QoL), mortality, falls and fracture, unscheduled medical reviews and hospital presentations (emergency department visits not leading to admission, unplanned hospital admissions). No significant differences between intervention and control groups were observed for these outcomes (Table 2). However, this RCT was acknowledged by the authors as likely underpowered to detect differences in these outcomes [43].

The impact of deprescribing on medication cost was investigated by Curtin et al. [43] which reported that deprescribing contributed to medication cost savings; at 3-month follow-up, mean monthly medication cost savings were significantly greater for the intervention group than the control group.

## Quality assessment

**Fig. 2:**
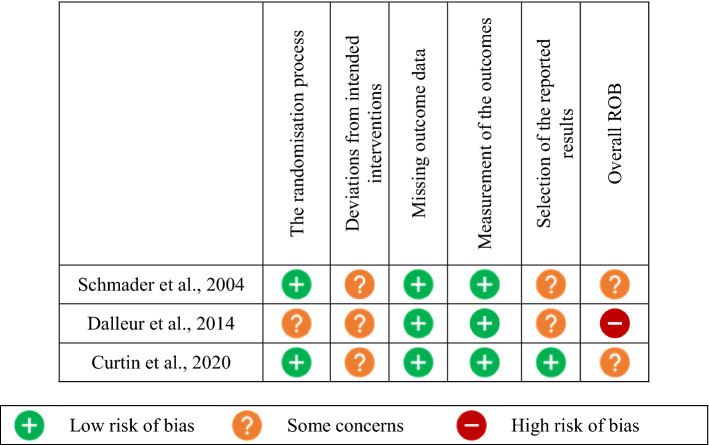
Risk-of-bias summary: review authors’ judgments about each risk of bias item for each included randomised controlled trial. (+) Low risk of bias; (−) High risk of bias; (?) Some concerns of bias

Risk of bias was assessed for prescribing appropriateness-related outcomes using ROB 2.0; results are summarised in Fig. 2. Two of the included studies were judged as having ‘some concerns’ of bias [41, 43], and one was judged as having a ‘high-risk’ of bias [42]. One study was judged as having ‘some concerns’ of bias associated with the randomisation process due to lack of reporting of allocation concealment [42]. None of the included studies reported sufficient details of analysis to estimate the effect of analysis (intention to treat analysis); all three studies were judged as having ‘some concerns’ in the ‘deviations from intended interventions’ domain. Curtin et al., (2020) was registered in ClinicalTrials.gov; the data were analysed according to a pre-specified plan and the study was judged to be at ‘low risk of bias in the ‘selection of the reported results’ domain [43]. The other two studies were considered to have ‘some concerns’ in this domain [41, 42].

## Discussion

### Statement of key findings

This review highlights the paucity of evidence regarding the effectiveness of medicines optimisation interventions on outcomes for frail older inpatients in secondary and acute care settings; only three studies were included in this review. This is in line with findings of previous reviews which highlighted the lack of intervention studies aimed at improving appropriate polypharmacy in older people in general [49, 50]. Recruiting in acute settings is often challenging due to acute illness, relatively short life expectancy, discharge before recruitment and data collection can be completed, difficulty in of follow-up post discharge, and lack of interest in participation [51].

Despite the recognition of the importance of frailty screening and assessment among older adults [52–55], many articles were excluded from this review at both abstract screening and full article screening stages because frailty was not diagnosed using a validated instrument. Several validated, simple, and quick assessment instruments are available and feasible to use in acute care [56–60] including the Clinical Frailty Scale [61], PRISMA 7 [62] and the Edmonton Frail Scale [63]. However, a lack of consensus definition for frailty and absence of agreement between frailty assessment instruments has hindered the wide implementation of these tools [52, 64].

Our systematic review assessed the impact of medicines optimisation interventions on prescribing in frail older inpatients and found that these interventions improved prescribing by reducing the number of PIMs. A previous Cochrane review to examine the impact of interventions to improve the appropriateness of prescribing in older people, albeit not specific for frail older people, in all types of healthcare settings reported improvement in the appropriateness of prescribing [50]. A recent systematic review and meta-analysis of RCTs to investigate the impact of different types of medicines optimisation interventions among older people living in residential care facilities or nursing homes found a significant improvement in medication appropriateness as demonstrated by reductions in the number of PIMs and the MAI score [65].

Two of the included studies reported that deprescribing reduced the number of regular medicines and PIMs consumed by frail older people [42, 43], and one reported that deprescribing did not adversely affect the rate of hospitalisation or mortality [43]. This reflects similar findings reported in a recent systematic review of deprescribing for frail older people in all types of healthcare settings, which demonstrated the feasibility, safety, tolerability and effectiveness of deprescribing in reducing the number of prescribed medications and number of PIMs [33]. These findings are also consistent with the findings of several other systematic reviews that examine the impact of deprescribing among older people in primary and secondary care settings as well as community-dwelling older adults [66–68].

Schmader et al. addressed the impact of medicines optimisation interventions on all aspects of PIP, including underprescribing [41], with the other two studies focusing on reducing the number of prescribed medications without addressing underuse [42, 43]. This is consistent with the findings of the Cochrane review of interventions to improve the appropriate use of polypharmacy in older people in any healthcare setting, which highlighted a dearth of evidence that assesses underprescribing [50] despite the prevalence of underprescribing among older populations [17, 19, 20, 69]and its association with negative health outcomes including increased morbidity, disability, healthcare utilisation, costs, and mortality [18].

Our review also assessed the impact of medicines optimisation interventions on clinical outcomes including falls, fractures, hospital presentation, QoL and mortality. The Curtin et al. study examined these outcomes and reported no significant differences in any measured outcome, but was not powered to evaluate any of these clinical outcomes [43]., Previous reviews in different clinical settings examined the impact of different types of medicines optimisation interventions in older patients and found limited evidence to drive strong conclusions about the impact on clinical outcomes; similarly, the majority of studies were not powered to detect significant differences [50, 65, 66, 70–73]. This gap could be addressed through adequately powered studies designed to specifically evaluate any of these clinical outcomes. A recent systematic review of pharmacist-led interventions in older adults identified three RCTs out of 35 included studies which were adequately powered to detect differences in clinical outcomes. These RCTs found a significant association between a pharmacist-led intervention in older adults and a reduction in post-discharge hospital visits [74].

There is growing financial pressure on the healthcare system to meet the needs of older patients, particularly frail patients, to support them to maintain independent and healthier lifestyles [75, 76]. Consequently, cost-effectiveness data are required to increase the chances of successful implementation of any medicines optimisation service and to ensure effective resource utilisation. One study in this review examined the impact of deprescribing on medication costs and demonstrated a significant reduction [43]. This is in line with the findings of a previous systematic review and interventional studies conducted in different healthcare settings including nursing homes, intermediate and acute care settings, which found that medication review and deprescribing among older people generated substantial cost savings and a reduction in medication costs [33, 77–86]. However, the cost-effectiveness of medicines optimisation is still unclear and many reviews have highlighted the paucity of evidence regarding the economic impact of these interventions [87–89].

## Strengths and weaknesses

This is the first systematic review to specifically investigate the impact of medicines optimisation interventions on prescribing and clinical outcomes for hospitalised frail older patients. Several systematic reviews have examined the impact of medicines optimisation interventions on PIMs and clinical outcomes in older patients in heterogeneous settings. However, the included studies did not explicitly target frail patients in whom frailty was assessed using a validated frailty tool [33, 50, 65, 66, 70, 90, 91]. A further strength of this systematic review was the rigorous methodology employed; a comprehensive search of large databases and trial registries was undertaken to include all types of studies and ongoing studies, with two reviewers independently screening all retrieved studies for inclusion. However, several limitations must be acknowledged when interpreting the findings of this systematic review. Explicitly including studies published in English may have led to language bias. Due to significant heterogeneity in intervention type and outcome measures, it was not possible to conduct a meta-analysis, and robust conclusions on efficacy by intervention type could not be made due to the limited number of included studies. Furthermore, the narrative synthesis presented should be treated with caution due to the small number and low quality of the included studies; none of the included studies were judged to be at low risk of bias and one was judged to be at ‘high risk’ of bias.

## Interpretation and further research

Medicines optimisation is a safe, feasible, and effective approach to improve the appropriateness of prescribing in the acute care settings. High-quality studies are needed to outline the cost-effectiveness and impact of medicines optimisation for frail hospitalised older patients. Strategies that facilitate the identification of frail patients in hospitals and the recruitment of these patients in clinical trials should also be implemented.

## Conclusion

This systematic review highlights the paucity and the low quality of evidence examining the impact of medicines optimisation on quality of prescribing and clinical outcomes for frail older inpatients which limits our ability to draw robust conclusions. It suggests that medicines optimisation interventions may improve prescribing appropriateness in frail older inpatients. However, although their impact on frail patients’ clinical outcomes is unclear, these interventions seem to be safe and feasible for implementation in acute settings.

## Supplementary Information

Below is the link to the electronic supplementary material.

Supplementary file1 (DOCX 23 kb)

Supplementary file2 (DOCX 36 kb)

Supplementary file3 (DOCX 28 kb)

## Data Availability

Data derived from sources in the public domain. All data were obtained from published papers.
