# Supplementary material for: Interventions to improve medicines optimisation in frail older patients in secondary and acute care settings: a systematic review of randomised controlled trials and non-randomised studies
Source: Int J Clin Pharm. 2021 Nov 20;44(1):15–26. doi: 10.1007/s11096-021-01354-8 (PMC8866367; doi:10.1007/s11096-021-01354-8)
Supplement: Supplementary file 2 — Supplementary file2 (DOCX 36 kb) [file 11096_2021_1354_MOESM2_ESM.docx]

**Supplementary Material 2: Search Strategy**

**Medline search strategy**

|  | exp Hospitals/ |
| --- | --- |
|  | hospital*.mp. |
|  | (acute adj1 care).mp. |
|  | Secondary Care/ |
|  | (secondary adj1 care).mp. |
|  | Intensive Care Units/ |
|  | ICU*.mp. |
|  | (Intensive adj1 care adj1 unit).mp. |
|  | Emergency Service, Hospital/ |
|  | Emergency adj1 Department).mp. |
|  | (Accident and Emergency).mp. |
|  | accident adj1 Emergency).mp. |
|  | Ambulatory Care Facilities/ |
|  | (outpatients adj1 clinic).mp. |
|  | exp Ambulances/ |
|  | ambulance.mp. |
|  | paramedic.mp. |
|  | acute care setting.mp. |
|  | ED.mp. |
|  | outpatient clinic.mp. |
|  | 1 or 2 or 3 or 4 or 5or 6 or 7 or 8 or 9 or 10 or 11 or 12 or 13 or 14 or 15 or 16 or 17 or 18 or 19 or 20 |
|  | Frail Elderly/ or Frailty/ |
|  | (frail adj1 elderly).mp. |
|  | (frail adj1 older adj1 adult).mp. |
|  | (frail adj1 older adj1 person).mp. |
|  | (frail adj1 older adj1 people).mp. |
|  | frail adj1 aged).mp. |
|  | (frail adj1 geriatric).mp. |
|  | (frail adj1 old adj1 age).mp. |
|  | 22 or 23 or 24 or 25 or 26 or 27 or 28 or 29 |
|  | exp. Medication Therapy Management/ |
|  | (frail adj1 elderly).mp. |
|  | (frail adj1 old adj1 age).mp. |
|  | 22 or 23 or 24 or 25 or 26 or 27 or 28 or 29 |
|  | Medication Therapy Management/ |
|  | (medicine* adj1 management).mp. |
|  | (medication adj1 management).mp. |
|  | (medicine* adj1 optimi?ation).mp. |
|  | (medication adj1 optimi?ation).mp. |
|  | pharmaceutical services/ or pharmacy service, hospital/ |
|  | (pharmaceutical adj1 care).mp. |
|  | "Drug Utilization Review"/ or Medication Reconciliation/ |
|  | (medication adj1 review).mp. |
|  | (medicine* adj1 review).mp. |
|  | prescribing.mp. |
|  | (prescribing adj1 guideline*).mp. |
|  | deprescribing.mp. or Deprescriptions/ |
|  | inappropriate Prescribing/ |
|  | (inappropriate adj1 prescribing).mp. |
|  | (appropriate adj1 prescribing).mp. |
|  | (over adj1 prescribing).mp. |
|  | Medication Adherence/ |
|  | (patient adj1 medication adj1 compliance).mp. |
|  | (medication adj1 nonadherence).mp. |
|  | (drug adj1 regime adj1 review).mp. |
|  | (drug adj1 utili?ation).mp. |
|  | (improv* adj1 prescrib*).mp. |
|  | (improv* adj1 prescription*).mp. |
|  | (medication adj1 adherance).mp. |
|  | underprescribing.mp. |
|  | overprescribing.mp. |
|  | (under adj1 prescribing).mp. |
|  | 31 or 32 or 33 or 34 or 35 or 36 or 37 or 38 or 39 or 38 or 39 or 40 or 41 or 42 or 43 or 44 or 45 or 46 or 47 or 48 or49 or 50 or 51 or 52 or 53 or 54 or 55 or 56 or 57 or 68 |
|  | 21 and 30 and 59 |

‘exp’=explode; ‘adj’= two adjacent terms; ‘adj1’=a maximum of one word between two adjacent terms; ‘adj2’=a maximum of two words between two adjacent terms; ‘*’=truncated term; ‘?’=extra letter; ‘mp’=title, abstract, original title, name of substance word, subject heading word, keyword heading word, protocol supplementary concept word, rare disease supplementary concept word, unique identifier, synonyms

**Embase search strategy**

|  | exp Hospitals/ |
| --- | --- |
|  | hospital*.mp. |
|  | (acute adj1 care).mp. |
|  | Secondary Care/ |
|  | (secondary adj1 care).mp. |
|  | Intensive Care Units/ |
|  | ICU*.mp. |
|  | (Intensive adj1 care adj1 unit).mp. |
|  | Emergency Service, Hospital/ |
|  | Emergency adj1 Department).mp. |
|  | (Accident and Emergency).mp. |
|  | accident adj1 Emergency).mp. |
|  | Ambulatory Care Facilities/ |
|  | (outpatients adj1 clinic).mp. |
|  | exp Ambulances/ |
|  | ambulance.mp. |
|  | paramedic.mp. |
|  | acute care setting.mp. |
|  | ED.mp. |
|  | outpatient clinic.mp. |
|  | 1 or 2 or 3 or 4 or 5or 6 or 7 or 8 or 9 or 10 or 11 or 12 or 13 or 14 or 15 or 16 or 17 or 18 or 19 or 20 |
|  | Frail Elderly/ or Frailty/ |
|  | (frail adj1 elderly).mp. |
|  | (frail adj1 older adj1 adult).mp. |
|  | (frail adj1 older adj1 person).mp. |
|  | (frail adj1 older adj1 people).mp. |
|  | frail adj1 aged).mp. |
|  | (frail adj1 geriatric).mp. |
|  | (frail adj1 old adj1 age).mp. |
|  | 22 or 23 or 24 or 25 or 26 or 27 or 28 or 29 |
|  | exp. Medication Therapy Management/ |
|  | (frail adj1 elderly).mp. |
|  | (frail adj1 old adj1 age).mp. |
|  | 22 or 23 or 24 or 25 or 26 or 27 or 28 or 29 |
|  | Medication Therapy Management/ |
|  | (medicine* adj1 management).mp. |
|  | (medication adj1 management).mp. |
|  | (medicine* adj1 optimi?ation).mp. |
|  | (medication adj1 optimi?ation).mp. |
|  | pharmaceutical services/ or pharmacy service, hospital/ |
|  | (pharmaceutical adj1 care).mp. |
|  | "Drug Utilization Review"/ or Medication Reconciliation/ |
|  | (medication adj1 review).mp. |
|  | (medicine* adj1 review).mp. |
|  | prescribing.mp. |
|  | (prescribing adj1 guideline*).mp. |
|  | deprescribing.mp. or Deprescriptions/ |
|  | inappropriate Prescribing/ |
|  | (inappropriate adj1 prescribing).mp. |
|  | (appropriate adj1 prescribing).mp. |
|  | (over adj1 prescribing).mp. |
|  | Medication Adherence/ |
|  | (patient adj1 medication adj1 compliance).mp. |
|  | (medication adj1 nonadherence).mp. |
|  | (drug adj1 regime adj1 review).mp. |
|  | (drug adj1 utili?ation).mp. |
|  | (improv* adj1 prescrib*).mp. |
|  | (improv* adj1 prescription*).mp. |
|  | (medication adj1 adherance).mp. |
|  | underprescribing.mp. |
|  | overprescribing.mp. |
|  | (under adj1 prescribing).mp. |
|  | 31 or 32 or 33 or 34 or 35 or 36 or 37 or 38 or 39 or 38 or 39 or 40 or 41 or 42 or 43 or 44 or 45 or 46 or 47 or 48 or49 or 50 or 51 or 52 or 53 or 54 or 55 or 56 or 57 or 68 |
|  | 21 and 30 and 59 |

‘exp’=explode; ‘adj’= two adjacent terms; ‘adj1’=a maximum of one word between two adjacent terms; ‘adj2’=a maximum of two words between two adjacent terms; ‘*’=truncated term; ‘?’=extra letter; ‘mp’=title, abstract, original title, name of substance word, subject heading word, keyword heading word, protocol supplementary concept word, rare disease supplementary concept word, unique identifier, synonyms

**International Pharmaceutical Abstracts** **(IPA) search strategy**

|  | exp Hospitals/ |
| --- | --- |
|  | hospital*.mp. |
|  | (acute adj1 care).mp. |
|  | Secondary Care/ |
|  | (secondary adj1 care).mp. |
|  | Intensive Care Units/ |
|  | ICU*.mp. |
|  | (Intensive adj1 care adj1 unit).mp. |
|  | Emergency Service, Hospital/ |
|  | Emergency adj1 Department).mp. |
|  | (Accident and Emergency).mp. |
|  | accident adj1 Emergency).mp. |
|  | Ambulatory Care Facilities/ |
|  | (outpatients adj1 clinic).mp. |
|  | exp Ambulances/ |
|  | ambulance.mp. |
|  | paramedic.mp. |
|  | acute care setting.mp. |
|  | ED.mp. |
|  | outpatient clinic.mp. |
|  | 1 or 2 or 3 or 4 or 5or 6 or 7 or 8 or 9 or 10 or 11 or 12 or 13 or 14 or 15 or 16 or 17 or 18 or 19 or 20 |
|  | Frail Elderly/ or Frailty/ |
|  | (frail adj1 elderly).mp. |
|  | (frail adj1 older adj1 adult).mp. |
|  | (frail adj1 older adj1 person).mp. |
|  | (frail adj1 older adj1 people).mp. |
|  | frail adj1 aged).mp. |
|  | (frail adj1 geriatric).mp. |
|  | (frail adj1 old adj1 age).mp. |
|  | 22 or 23 or 24 or 25 or 26 or 27 or 28 or 29 |
|  | exp. Medication Therapy Management/ |
|  | (frail adj1 elderly).mp. |
|  | (frail adj1 old adj1 age).mp. |
|  | 22 or 23 or 24 or 25 or 26 or 27 or 28 or 29 |
|  | Medication Therapy Management/ |
|  | (medicine* adj1 management).mp. |
|  | (medication adj1 management).mp. |
|  | (medicine* adj1 optimi?ation).mp. |
|  | (medication adj1 optimi?ation).mp. |
|  | pharmaceutical services/ or pharmacy service, hospital/ |
|  | (pharmaceutical adj1 care).mp. |
|  | "Drug Utilization Review"/ or Medication Reconciliation/ |
|  | (medication adj1 review).mp. |
|  | (medicine* adj1 review).mp. |
|  | prescribing.mp. |
|  | (prescribing adj1 guideline*).mp. |
|  | deprescribing.mp. or Deprescriptions/ |
|  | inappropriate Prescribing/ |
|  | (inappropriate adj1 prescribing).mp. |
|  | (appropriate adj1 prescribing).mp. |
|  | (over adj1 prescribing).mp. |
|  | medication adherence.mp. or medication compliance/ |
|  | (patient adj1 medication adj1 compliance).mp. |
|  | (medication adj1 nonadherence).mp. |
|  | (drug adj1 regime adj1 review).mp. |
|  | (drug adj1 utili?ation).mp. |
|  | (improv* adj1 prescrib*).mp. |
|  | (improv* adj1 prescription*).mp. |
|  | (medication adj1 adherance).mp. |
|  | underprescribing.mp. |
|  | overprescribing.mp. |
|  | (under adj1 prescribing).mp. |
|  | 31 or 32 or 33 or 34 o 35 or 36 or 37 or 38 or 39 or 38 or 39 or 40 or 41 or 42 or 43 or 44 or 45 or 46 or 47 or 48 or49 or 50 or 51 or 52 or 53 or 54 or 55 or 56 or 57 or 68 |
|  | 21 and 30 and 59 |

‘exp’=explode; ‘adj’= two adjacent terms; ‘adj1’=a maximum of one word between two adjacent terms; ‘adj2’=a maximum of two words between two adjacent terms; ‘*’=truncated term; ‘?’=extra letter; ‘mp’=title, abstract, original title, name of substance word, subject heading word, keyword heading word, protocol supplementary concept word, rare disease supplementary concept word, unique identifier, synonyms

**Cumulative Index to Nursing and Allied Health Literature Plus** (**CINAHL Plus)** **search strategy**

| S1 | "hospital" |
| --- | --- |
| S2 | hospital* |
| S3 | "acute care" |
| S4 | acute N1 care |
| S5 | acute care |
| S6 | "secondary care" |
| S7 | secondary care |
| S8 | secondary N1 care |
| S9 | "intensive care unit" OR (MH "Intensive Care Units") |
| S10 | intensive N1 care N1 unit |
| S11 | intensive care unit |
| S12 | (MH "Acute Care") |
| S13 | (MH "Secondary Health Care") |
| S14 | (MH "Intensive Care Units") |
| S15 | intensive N1 care N1 unit |
| S16 | intensive care unit |
| S17 | (MH "Emergency Service") |
| S18 | emergency service* |
| S19 | emergency N1 services |
| S20 | "accident and emergency" |
| S21 | accident and emergency |
| S22 | accident N1 and N1 emergency |
| S23 | (MH "Ambulatory Care") OR (MH "Ambulatory Care Facilities") |
| S24 | ambulatory care |
| S25 | ambulatory N1 care |
| S26 | outpatient N1 clinic |
| S27 | "outpatient clinic" OR (MH  "Outpatient Service") |
| S28 | outpatient clinic |
| S29 | paramedic |
| S30 | "paramedic" |
| S31 | ambulance |
| S32 | (MH "Ambulances") |
| S33 | S1 OR S2 OR S3 OR S4 OR S5 OR S6 OR S7 OR S8 OR S9 OR S10 OR S11 OR S12 OR S13 OR S14 OR S15 OR S16 OR S17 OR S18 OR S19 OR S20 OR S21 OR S22 OR S23 OR S24 OR S25 OR S26 OR S27 OR S28 OR S29 OR S30 OR S31 OR S32 OR S33 |
| S34 | under N1 prescribing |
| S35 | "underprescribing" |
| S36 | improve* N1 prescription* |
| S37 | improve* N1 prescrib* |
| S38 | drug N1 utili?ation |
| S39 | drug N1 regime N1 review |
| S40 | medication N1 nonadherence |
| S41 | medication N1 compliance |
| S42 | medication N1 adherence |
| S43 | (MH "Medication Compliance") OR "medication adherence" |
| S44 | "overprescribing" |
| S45 | over N1 prescribing |
| S46 | appropriate N1 prescribing |
| S47 | inappropriate N1 prescribing |
| S48 | deprescribing |
| S50 | "deprescribing" |
| S51 | prescribing N1 guideline* |
| S52 | (MH "Inappropriate Prescribing") OR "prescribing" |
| S53 | drug N1 utilli?ation N1 review |
| S54 | (MH "Medication Reconciliation") |
| S55 | medicine* N1 review |
| S56 | medication N1 review |
| S57 | "medication review" |
| S58 | (MH "Drug Utilization") OR "drug utilization review" |
| S59 | "pharmaceutical services" |
| S60 | pharmaceutical N1 care |
| S61 | pharmaceutical care |
| S62 | medication N1 optimi? ation |
| S63 | medicine* N1 optimi?ation |
| S64 | medication N1 management |
| S65 | medicine* N1 management |
| S66 | "medication optimisation" |
| S67 | medication N1 therapy N1 management |
| S68 | (MH "Medication Management") OR "medication therapy management" |
| S69 | S86 OR S67 OR S66 OR S65 OR S64 OR S63 OR S62 OR S61 OR S60 OR S59 OR S58 OR S57 OR S56 OR S55 OR S54 OR S53 OR S52 OR S51 OR S50 OR S49 OR S48 OR S47 OR S46 OR S45 OR S44 OR S43 OR S42 OR S41 OR S40 OR S39 OR S38 OR S37 OR S36 OR S35 OR S34 |
| S70 | Frail N1 old N1 age |
| S71 | frail N1 geriatric |
| S72 | frail aged |
| S73 | Frail N1 aged |
| S74 | frail N1 older N1 person |
| S75 | Frail N1 older N1 adult |
| S76 | "frail older adults" |
| S77 | frail N1 elderly |
| S78 | frail elderly |
| S79 | (MH "Frail Elderly") |
| S80 | S70 Or S71 OR S72 OR S73 OR S74 OR S75 OR S76 OR S78 OR S79 |
| S81 | S33 AND S69 AND S80 |

‘MH’= subject heading has been searched; ‘N1’= a maximum of one words between two adjacent terms; ‘N2’= a maximum of two words between two adjacent terms; ‘+’ = the subject heading has been exploded; ‘*’=truncated term

**Scopus search strategy**

("hospital" OR "acute care" OR "secondary care" OR "Intensive care unit" OR "emergency department" OR "accident and emergency" OR "ambula*" O R "outpatients clinic" OR "paramedic*" OR "intermediate care") AND ( 'frail'' OR ''frailty'' OR "frail elderly" OR "Frail older adult" OR "frail older person" OR "frail older people" OR "frail aged" OR "frail geriatric" OR "Frail old age" ) AND ( "medication therapy management" OR "medicine* management" OR "medication management" OR "medicine* optimi?ation" OR "medication optimi?ation" OR "pharmaceutical care" OR "pharmaceutical service*" OR "Drug utili?ation" OR "medication review" OR "medicine* review" OR "prescribing" OR " inappropriate pr escribing" OR "appropriate pr escribing" OR "overprescribing" OR "medication adherence" OR "medication non adherence" OR "patient medication compliance" OR "drug regime review" OR "drug utili?ation" OR "improv* prescrib*" OR "improv* prescription*" OR "underprescribing" OR ''compliance'' OR ''deprescribing'' ) AND ( LIMIT-TO ( LANGUAGE , "English" ) )

‘*’=truncated term

**Web of Science search strategy**

TS=("hospital*" or "acute care" or "secondary care" or "intensive care" or "emergency department" or "accident and emergency" or "ambula*" or "paramedic" or "outpatient clinic" or "intermediate care") AND TS=(''frail'' or ''frailty'' or "frail elderly" or "frail older adult" or "frail older person" or "frail older people" or "frail aged" or "frail geriatric" or "frail old age") AND TS=("medication therapy management" or "medicine* management" or "medication management" or "medicine* optimi?ation" or "medication optimi?ation" or "pharmaceutical care" or "pharmaceutical Services" or "Drug utili?ation" or "medicine* review" or "medication review" or "prescribing" or "prescribing guideline*" or "deprescription*" or "inappropriate prescribing" or "appropriate prescribing" or "over prescribing" or "medication adherence" or "medication nonadherence" or "medication non adherence" or "patient medication compliance" or "drug regime review" or "improv* prescrib*" or "improve* prescription" or"medication management" or "medicine* optimi?ation" or "medication optimi?ation" or "pharmaceutical care" or "pharmaceutical Services" or "Drug utili?ation" or "medicine* review" or "medication review" or "prescribing" or "prescribing guideline*" or "deprescribing" or "deprescription*" or "inappropriate prescribing" or "appropriate prescribing" or "over prescribing" or "medication adherence" or "medication nonadherence" or "medication non adherence" or "patient medication compliance" or "drug regime review" or "improv* prescrib*" or "improve* prescription" or ''underprescribing'') Refined by: LANGUAGES: ( ENGLISH )

**Cochrane Central Register of Controlled Trials and Cochrane Library**

("hospital" or "acute care" or "secondary care" or "Intensive care unit" or "emergency department" or "accident and emergency" or "ambula*" or "outpatients clinic" or "paramedic*" or ''intermediate care'') AND ("frail elderly" OR "Frail older adult" OR "frail older person" OR "frail older people" OR "frail aged" OR "frail geriatric" OR "Frail old age") AND ("medication therapy management" or '' medication compliance'' or "medicine* management" or "medication management" or "medicine* optimi?ation" or "medication optimi?ation" or ''drug utili?ation" or "improv* prescrib*" or "improv* prescription*" or ''underprescribing'' or "medicine* review" or "prescribing" or ''inappropriate prescribing" or "appropriate prescribing" or "overprescribing" or "medication adherence" or "medication non adherence" or "patient medication compliance" or "drug regime review" or "pharmaceutical care" or "pharmaceutical service*" or "Drug utili?ation" or "medication review")

‘*’=truncated term

**Clinical trials.gov**

**(Frailty/ frail older people and all studies)**

| “medication therapy management” | “patient medication compliance” |
| --- | --- |
| “medication management” | “medication compliance” |
| “medication review” | “medicine adherence” |
| “drug regime review” | “medication adherence” |
| “Medicine management” | “medication non adherence” |
| “Medicine optimization” | “drug utilization” |
| “medication optimization” | “medicine safety” |
| “Inappropriate prescribing” | “pharmaceutical care” |
| “appropriate prescribing” | “pharmaceutical service” |
| “overprescribing” | “improve prescribing” |
| “underprescribing” | “deprescribing” |

**International Clinical Trials Platform and Research Registry**

**(Frail/Frailty)**

| “medication therapy management” | “patient medication compliance” |
| --- | --- |
| “medication management” | “medication compliance” |
| “medication review” | “medicine adherence” |
| “drug regime review” | “medication adherence” |
| “Medicine management” | “medication non adherence” |
| “Medicine optimization” | “drug utilization” |
| “medication optimization” | “medicine safety” |
| “Inappropriate prescribing” | “pharmaceutical care” |
