# Supplementary material for: Interventions to improve medicines optimisation in frail older patients in secondary and acute care settings: a systematic review of randomised controlled trials and non-randomised studies
Source: Int J Clin Pharm. 2021 Nov 20;44(1):15–26. doi: 10.1007/s11096-021-01354-8 (PMC8866367; doi:10.1007/s11096-021-01354-8)
Supplement: Supplementary file 3 — Supplementary file3 (DOCX 28 kb) [file 11096_2021_1354_MOESM3_ESM.docx]

**Supplementary Material 3 Summary of excluded studies and reasons for exclusion**

| **Reference** | **Reason for exclusion** |
| --- | --- |
| **Full text articles** |  |
| Åhlund K, Bäck M, Öberg B, Ekerstad N. Effects of comprehensive geriatric assessment on physical fitness in an acute medical setting for frail elderly patients. Clin Interv Aging. 2017;12:1929–39. | Multicomponent intervention |
| Ekerstad N, Ivanoff SD, Landahl S, Östberg G, Johansson M, Andersson D, et al. Acute care of severely frail elderly patients in a CGA-unit is associated with less functional decline than conventional acute care. Clin Interv Aging. 2017;12:1239–49. | Multicomponent intervention |
| Ekerstad N, Karlson BW, Andersson D, Husberg M, Carlsson P, Heintz E, et al. Short-term resource utilization and cost-effectiveness of comprehensive geriatric assessment in acute hospital care for severely frail elderly patients. J Am Med Dir Assoc. 2018;19(10):871-878.e2. Available from: <https://doi.org/10.1016/j.jamda.2018.04.003> | Multicomponent intervention |
| Elston J, Gradinger F, Asthana S, Lilley-Woolnough C, Wroe S, Harman H, et al. Does a social prescribing “holistic” link-worker for older people with complex, multimorbidity improve well-being and frailty and reduce health and social care use and costs? A 12-month before-and-after evaluation. Prim Health Care Res Dev. 2019;20:e135. | 1.Not frail population as defined using a validated tool  2.Multicomponent intervention  Not focused on medicines optimisation  3.Age |
| Fairhall N, Aggar C, Kurrle SE, Sherrington C, Lord S, Lockwood K, et al. Frailty intervention trial (FIT). BMC Geriatr. 2008;8:1–10. | Multicomponent intervention |
| Graabæk T, Hedegaard U, Christensen MB, Clemmensen MH, Knudsen T, Aagaard L. Effect of a medicines management model on medication-related readmissions in older patients admitted to a medical acute admission unit—A randomized controlled trial. J Eval Clin Pract. 2019;25(1):88–96. | Not a frail population as defined using a validated tool |
| Marvin V, Ward E, Jubraj B, Bower M, Bovill I. Improving Pharmacists’ Targeting of Patients for Medication Review and Deprescription. Pharmacy. 2018;6(2):32. | Not an interventional study |
| Récoché I, Lebaudy C, Cool C, Sourdet S, Piau A, Lapeyre-Mestre M, et al. Potentially inappropriate prescribing in a population of frail elderly people. Int J Clin Pharm. 2017;39(1):113–9. | Not an interventional study - retrospective analysis of prescribing |
| Trentini M, Semeraro S, Motta M. Effectiveness of geriatric evaluation and care. one-year results of a multicenter randomized clinical trial. Aging Clin Exp Res. 2001;13(5):395–405. | 1. Not frail population as defined using a validated tool  2.No intervention related to any aspect medicines optimisation |
| **Clinical trials** |  |
| NCT02773914. Comprehensive geriatric assessment for frail older people. <https://clinicaltrials.gov/show/NCT02773914>. 2016;1–6. Available from: <https://www.cochranelibrary.com/central/doi/10.1002/central/CN-02043822/full%0Ahttps://www.cochranelibrary.com/central/doi/10.1002/central/CN-01581799/full>. | Multicomponent interventions |
| NCT02885337. Fit-Joint: getting fit for hip or knee replacement. <https://clinicaltrials.gov/show/NCT02885337>. 2016;1–8. Available from: <https://www.cochranelibrary.com/central/doi/10.1002/central/CN-01520529/full>. | Multicomponent interventions |
| NCT03719573. Geriatric assessment and intervention in older patients undergoing surgery for colon cancer. <https://clinicaltrials.gov/show/NCT03719573>. 2018;1–8. Available from: <https://www.cochranelibrary.com/central/doi/10.1002/central/CN-01700916/full>. | 1.Not using Frailty screening instrument  2.Multicomponent interventions; Not focused on medicines optimisation |
| NCT03751319. Geriatric assessment and intervention for older patients with frailty in the emergency department clinical trials. 2018. Available from: <https://clinicaltrials.gov/ct2/show/NCT03751319%0Ahttps://www.sbu.se/en/publications/responses-from-the-sbu-enquiry-service/clinical-frailty-scale-for-assessment-of-frailty-and-level-of-care-in-elderly-patients/>. | Multicomponent interventions |
| NCT01273116. In hospital care and welfare standard. clin trials. 2010;1–8. Available from: <https://clinicaltrials.gov/ct2/show/NCT01273116> | 1.Multicomponent interventions  2.Frailty screening tool was not used |
| NCT02715453. Intervention in frailty versus usual care in frail patients after an acute myocardial infarction. https://clinicaltrials.gov/show/NCT02715453; Available from: <https://www.cochranelibrary.com/central/doi/10.1002/central/CN-01556740/full?highlightAbstract=acute%7Cfrailti%7Cusual%7Cmyocardial%7Cin%7Cpatients%7Cinfarction%7Cfrail%7Cmyocardi%7Cacut%7Cintervent%7Cinfarct%7Cversus%7Cfrailty%7Cpatient%7Cafter%7Cintervention%7Ccare#.YHltvfzbvM8.mendeley>. | 1.Multi factorial interventions  Not focused on medicines optimisation  2.Prefrail was included |
| ISRCTN13051922. Breathe Plus:A trial to test the feasibility of including a comprehensive assessment at the start of lung rehabilitation for people living with chronic obstructive pulmonary disease and frailty. <http://www.who.int/trialsearch/Trial2.aspx?TrialID=ISRCTN13051922>. 2019;1–8. Available from: <https://www.cochranelibrary.com/central/doi/10.1002/central/CN-02070048/full>. | 1.Multicomponent interventions  2.Age of the participant |
| **Conference abstracts** |  |
| Camacho R, Sinclair A, Robson G, Gregson C. 115The feasibility of performing comprehensive geriatric assessment in the emergency department. Age Ageing. 2018;47(suppl. 2):ii25–39. | Not interventional study |
| Fernandez S. 7Essential pharmacy role in polypharmacy review and deprescribing for frail older patients. Age Ageing. 2019;48(Suppl. 2):ii1–10. | Unclear how frailty defined |
| Grion AM, Tinjala DD, Daragjati J, Pilotto A. Intervention to decrease the use of potentially inappropriate medication (PIM) among older people in two different settings. Eur Geriatr Med. 2013;4:S46. Available from: <http://dx.doi.org/10.1016/j.eurger.2013.07.150>. | Not a frail population defined using a validated tool |
| Hindmarsh J, O’Neil H, Cranmer P. 80Impact of frailty lead pharmacist on prescribing in frail patients on admission wards. Age Ageing. 2017;46(supp. 1):i1–22. | Unclear how frailty defined but all patients seen by the frailty team |
| Khor HM, Tan PJ, Saedon NI, Kamaruzzaman SB, Tan MP. 43Does a multifactorial interventional programme on falls prevention in older fallers improve frailty outcomes? Preliminary results from the malaysian falls assessment intervention trial (Myfait). Age Ageing. 2017;46(supp. 2):ii14–ii14. | Multicomponent intervention  Not focused on medicines optimisation |
| Kinahan C, Soomro N, Daniels F, Hussain W, Heery H. 43 Addressing polypharmacy in the frail older person - the WIDE (Wholistic, Integrated, Deprescribing, Evaluation) Review. Age Ageing. 2019;48(Supp. 3):iii17–65. | Conference abstract |
| Rosen S., McGalliard B, Shane R, Luong D, Tantipinichwong N, Amer K. Safe medication transitions in frailty patients. 2017;1(Supp. 1):973. | Unclear how frailty defined but patients described as "frailty patients" |
| Vince A, Pillay I, O’Reilly A, Cooney E, Maher J, Pender C, et al. 40 Using the variable indication of placement tool to drive de-prescribing in the frail older person in an interdisciplinary integrated team. Age Ageing. 2019;48(Supp. 3):iii17–65. | Unclear how frailty defined |
| Wildblood BAS, Clow L, Mohan AM, Deol P, Green E, Adams CA, et al. 68Targeted medication review to reduce anticholinergic burden on an acute frailty unit. Age Ageing. 2018;47(supp. 3):iii25–iii25. | Unclear how frailty defined but setting is an acute frailty unit |
| **Abstract (no available full article)** |  |
| Berard C, McCambridge C, Sourdet S, Piau A, Rouch L, Chicoulaa B, et al. Benzodiazepines and frail elderly people: how prescribing can be optimized?. Comment optimiser les Prescr benzodiazepines chez les patients ages fragiles ? [Internet]. 2018;16(4):359–66. Available from: <http://ovidsp.ovid.com/ovidweb.cgi?T=JS&PAGE=reference&D=med15&NEWS=N&AN=30378549>. | Full text not in English language. |
| Gill TH, Wasserman MR, Zolt RM. Pharmacist provided managed care in a geriatric continuity clinic. 1993;28:IPC-12. | Full text not available |
| Malik NA, Panagoda WK, Tan FYH, Pang WY. Frailty-guided management model for severely frail older adults in an acute hospital setting. Eur Geriatr Med. 2017;8(Supp. 1):S103. Available from: <http://ovidsp.ovid.com/ovidweb.cgi?T=JS&PAGE=reference&D=emed18&NEWS=N&AN=618532580>. | Multicomponent interventions  Not focused on medicines optimisation |
| McKeown W, Wright S, Alcorn M. 76Use of a Modified STOPPFrail Assessment to aid de-prescribing in a district general hospital. Age Ageing [Internet]. 2018 1;47(supp. 5):v13–60. Available from: <https://doi.org/10.1093/ageing/afy140.57>. | Conference presentation |
| Mosnier-Thoumas S, Videau M-N, Lafargue A, Martin-Latry K, Salles N. Benefit of the geriatric mobile unit’s intercession on the quality of medical prescriptions for the elderly: Past year assessment . Ann Pharm Fr. 2019;77(2):136–45. Available from: <https://www.scopus.com/inward/record.uri?eid=2-s2.0-85055746161&doi=10.1016%2Fj.pharma.2018.10.002&partnerID=40&md5=60be6d6d7543a42cc20eab8304c7d9cc>. | Full text not in English language. Also query whether tool used to define frailty |
| Torbati AA, Sinvani L, Kast C, Bianculli A, Wirostek S, Sharma A. Geriatric care model. J Am Geriatr Soc. 2018;66(Supp. 2):S108. Available from: <http://ovidsp.ovid.com/ovidweb.cgi?T=JS&PAGE=reference&D=emed19&NEWS=N&AN=622131118>. | Frailty not defined using tool |
| Trentini M, Semeraro S, Rossi E, Giannandrea E, Vanelli M, Pandiani G, et al. A multicenter randomized trial of comprehensive geriatric assessment and management: Experimental design, baseline data, and six-month preliminary results. Aging Clin Exp Res. 1995;7(3):224–33. Available from: http://link.springer.com/10.1007/BF03324320 | Multicomponent interventions Not focused on medicines optimisation  Not frail |
